# Supplementary material for: Hydroxy-octadecenoic acids instead of phorbol esters are responsible for the Jatropha curcas kernel cake’s toxicity
Source: Commun Biol. 2020 May 8;3:228. doi: 10.1038/s42003-020-0919-z (PMC7210109; doi:10.1038/s42003-020-0919-z)
Supplement: Supplementary file 1 — Supplementary Information [file 42003_2020_919_MOESM1_ESM.pdf]

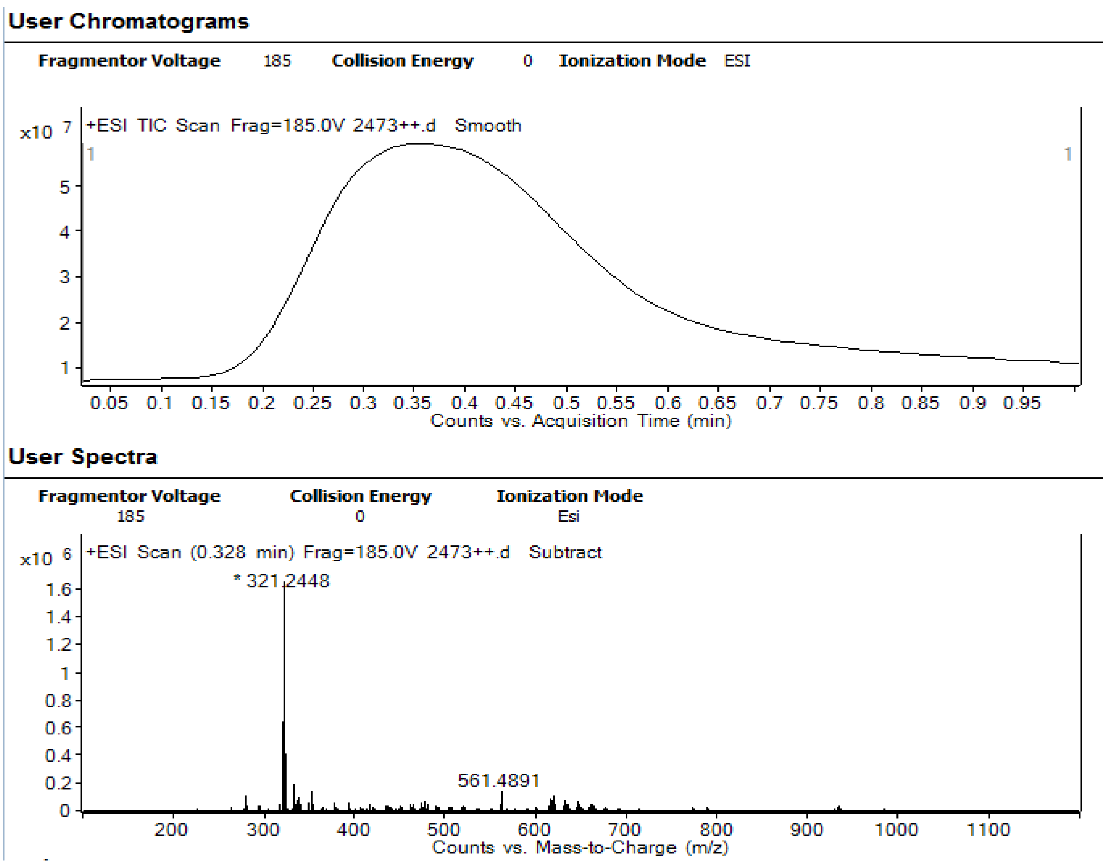

Supplementary Figure 1. The HR-ESI-MS of mixture 1 (hydroxy-octadecenoic acids).

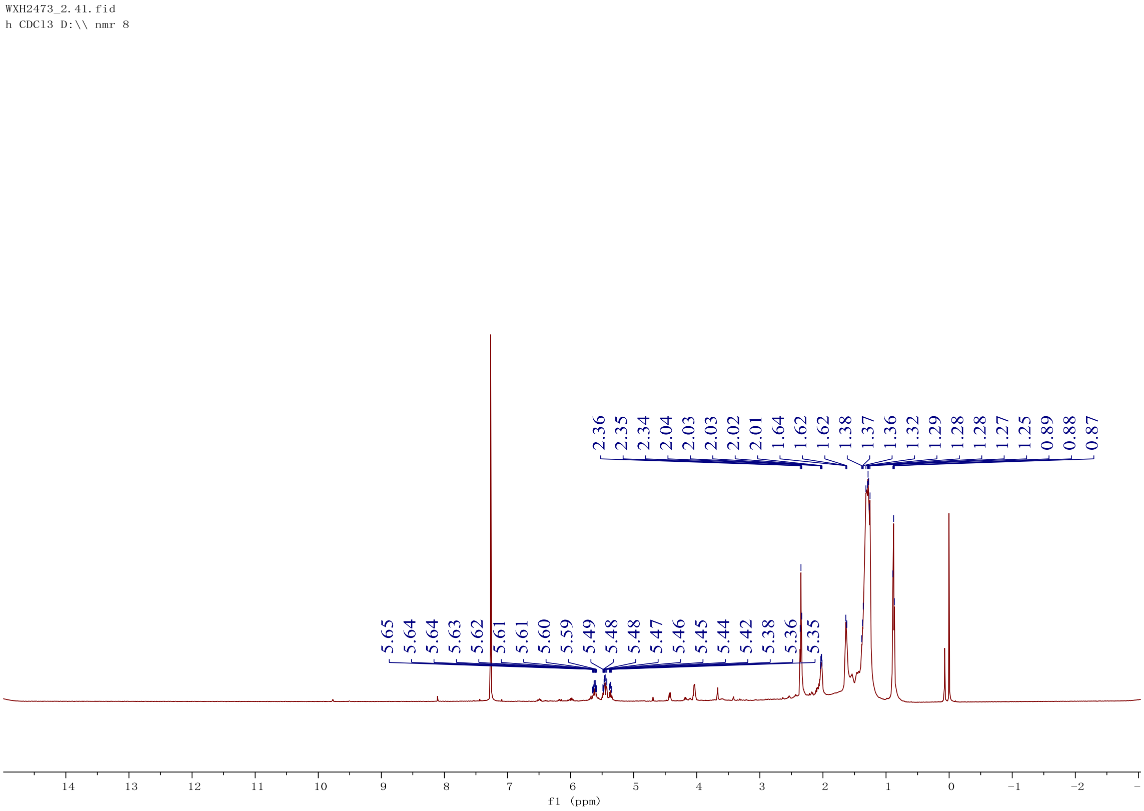

Supplementary Figure 2. 1H-NMR of Mixture 1 (hydroxy-octadecenoic acids).

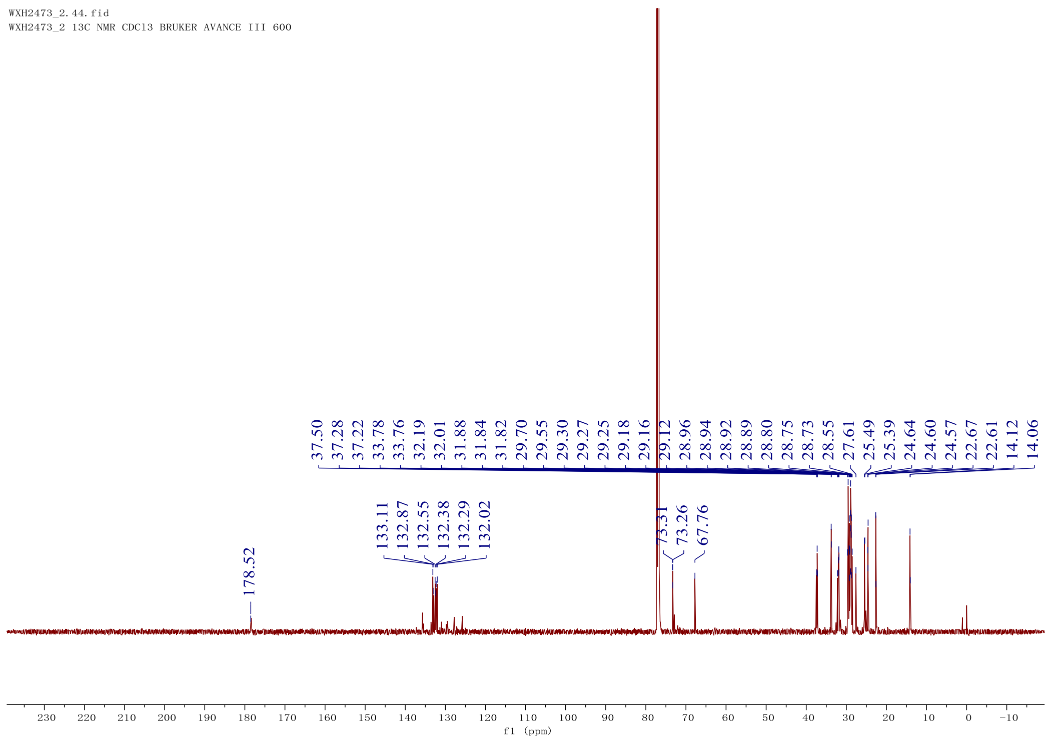

Supplementary Figure 3. 13C-NMR of Mixture 1 (hydroxy-octadecenoic acids).

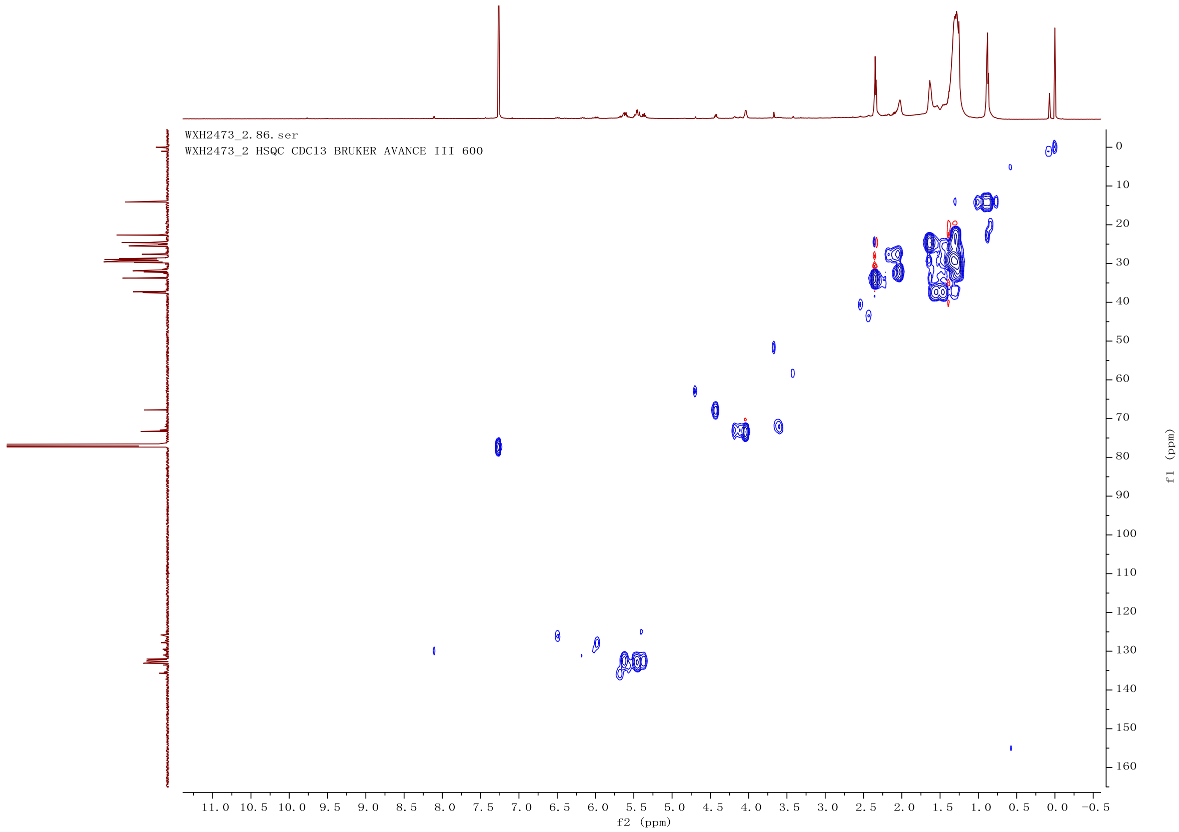

Supplementary Figure 4. HSQC of Mixture 1 (hydroxy-octadecenoic acids).

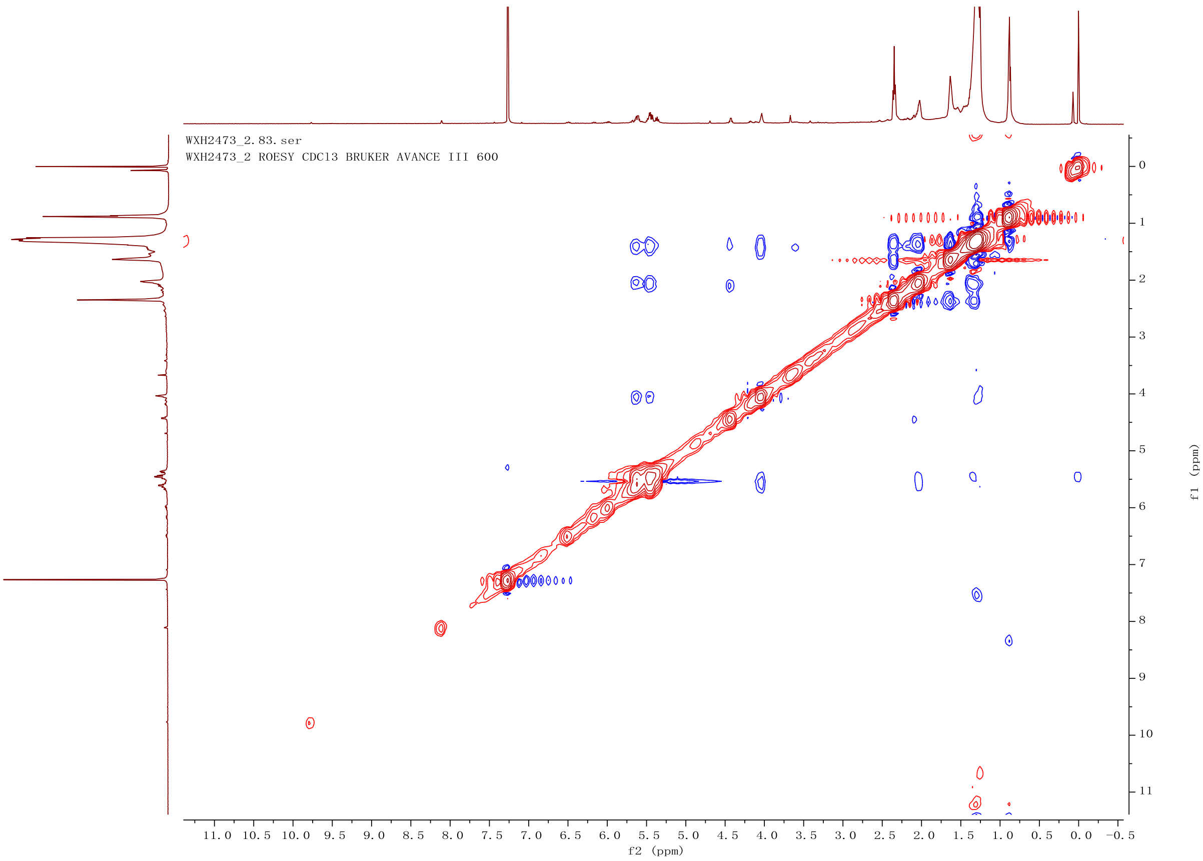

Supplementary Figure 5. ROESY of Mixture 1 (hydroxy-octadecenoic acids).

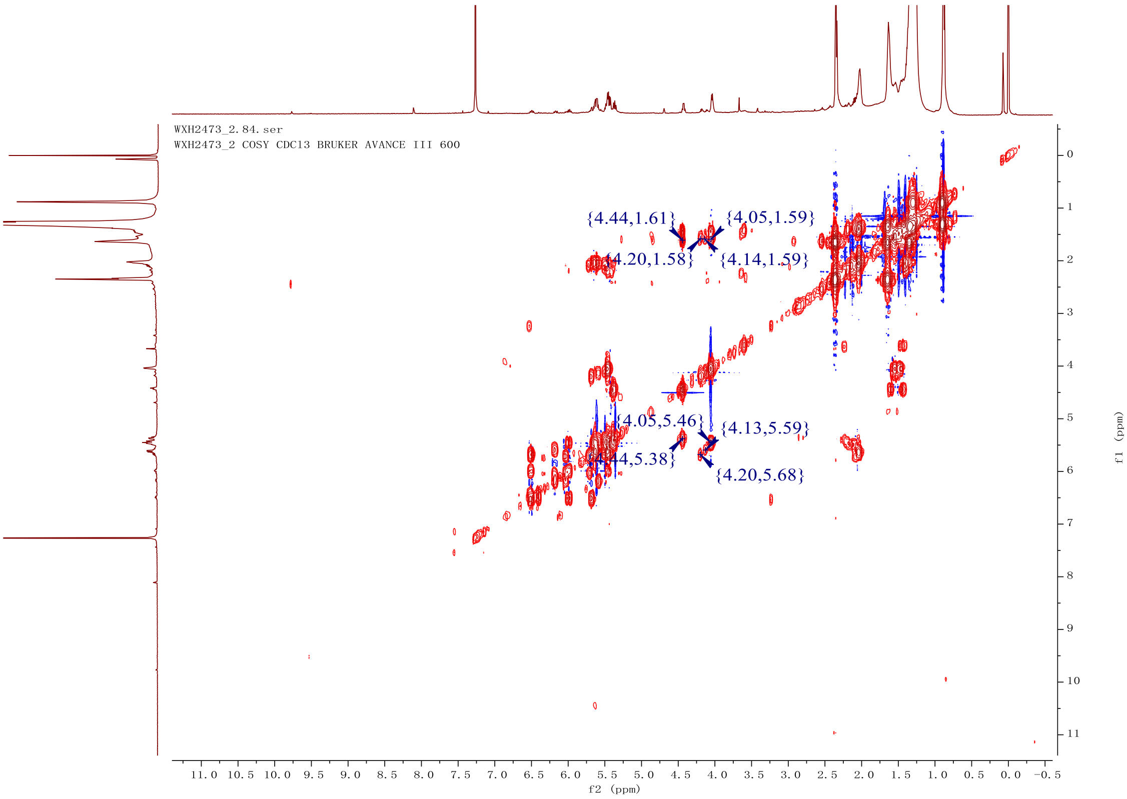

Supplementary Figure 6. 1H-1H COSY of Mixture 1 (hydroxy-octadecenoic acids).

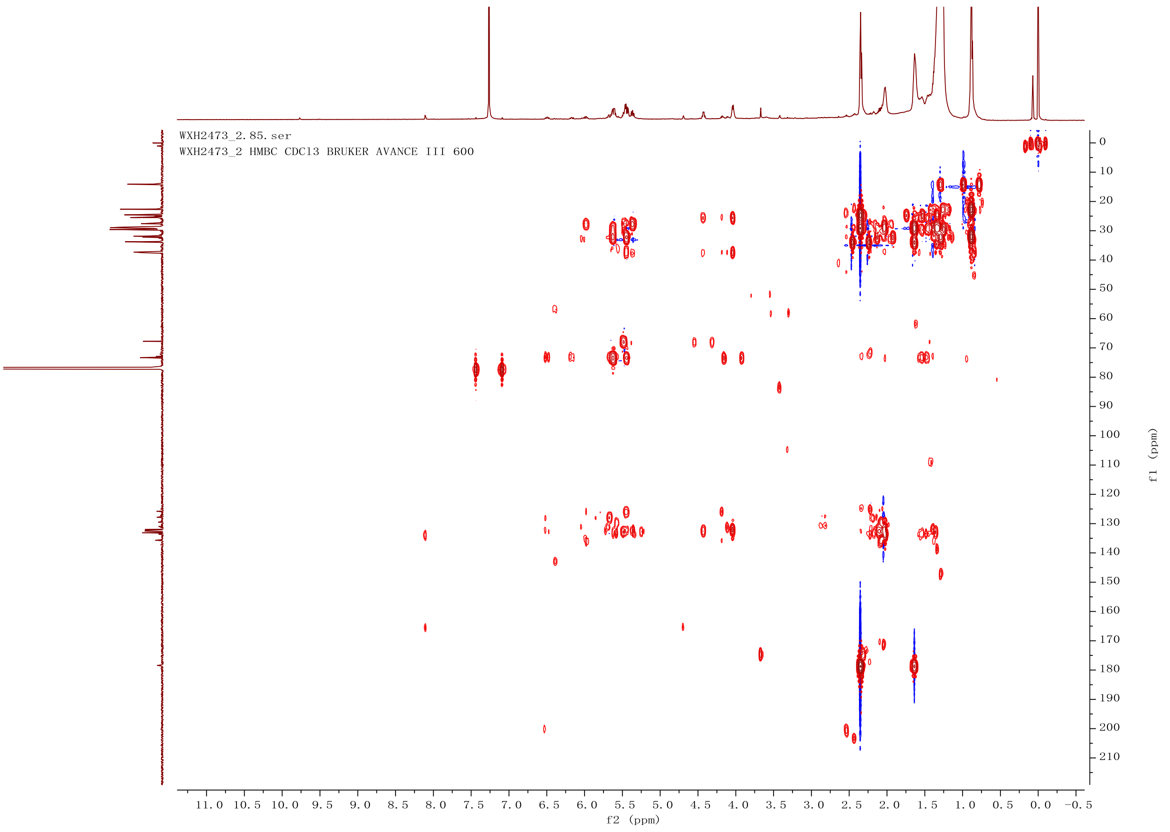

Supplementary Figure 7. HMBC of Mixture 1 (hydroxy-octadecenoic acids).

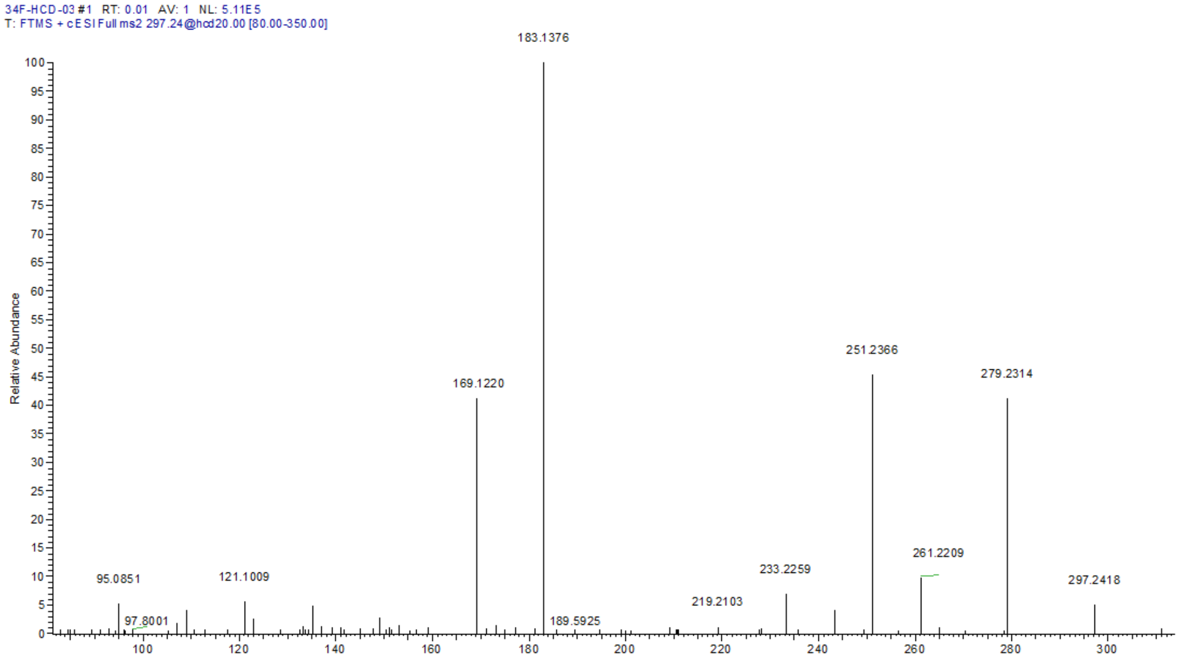

Supplementary Figure 8. The HR-ESI-MS of Mixture 2.

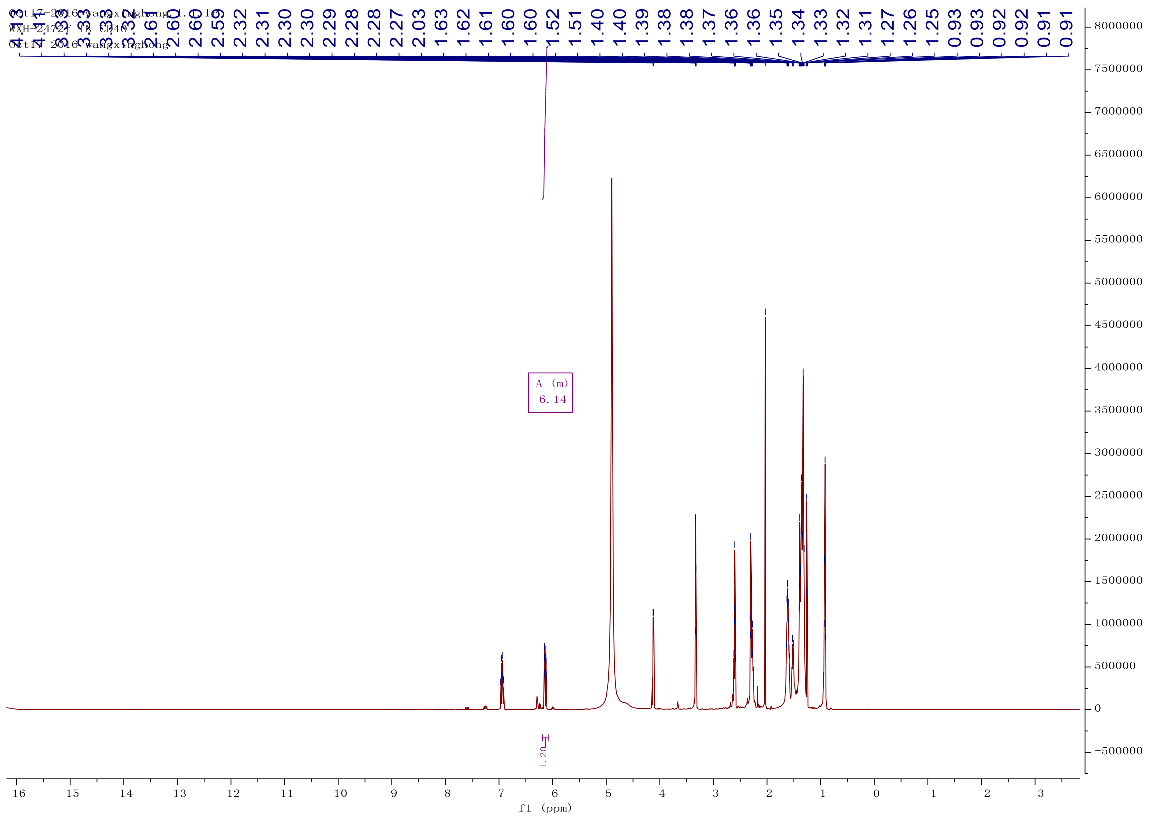

Supplementary Figure 9. 1H-NMR of Mixture 2.

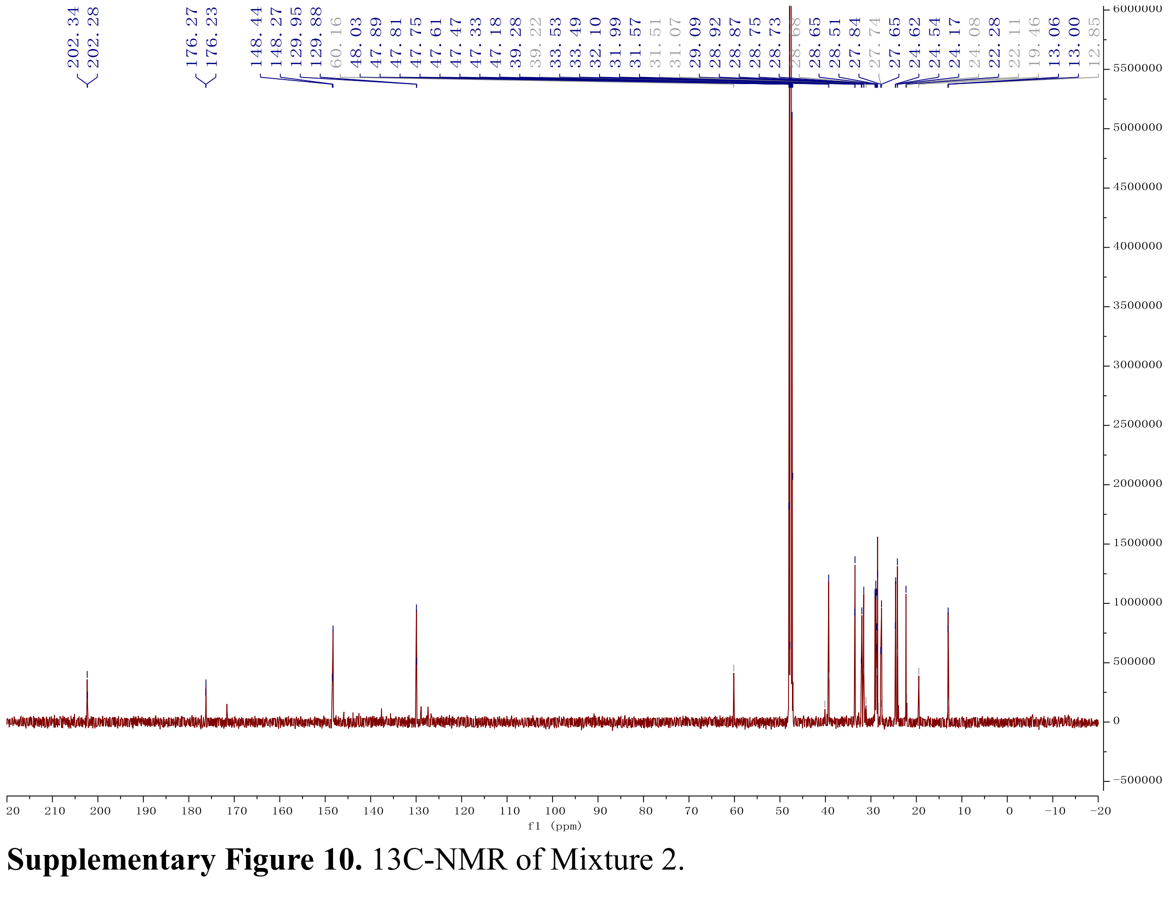

Supplementary Figure 10. 13C-NMR of Mixture 2.

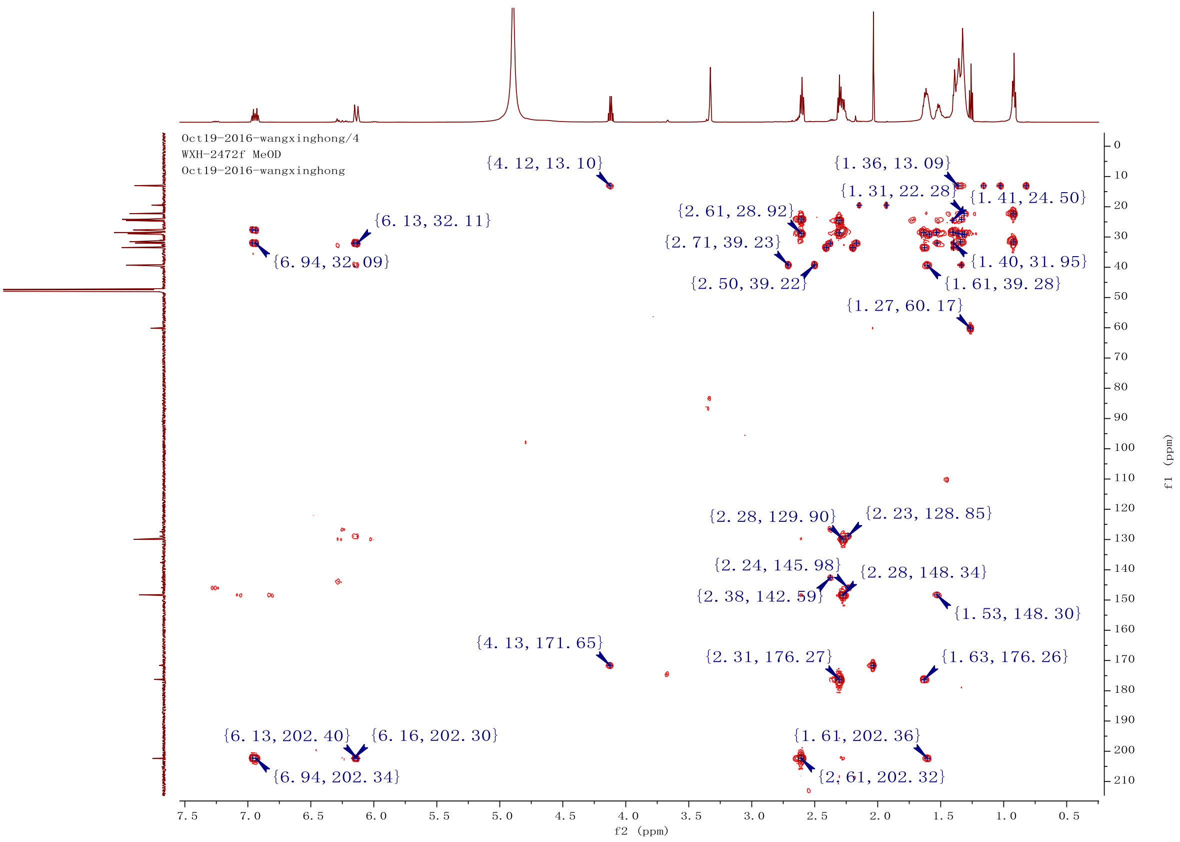

Supplementary Figure 11. HMBC of Mixture 2.

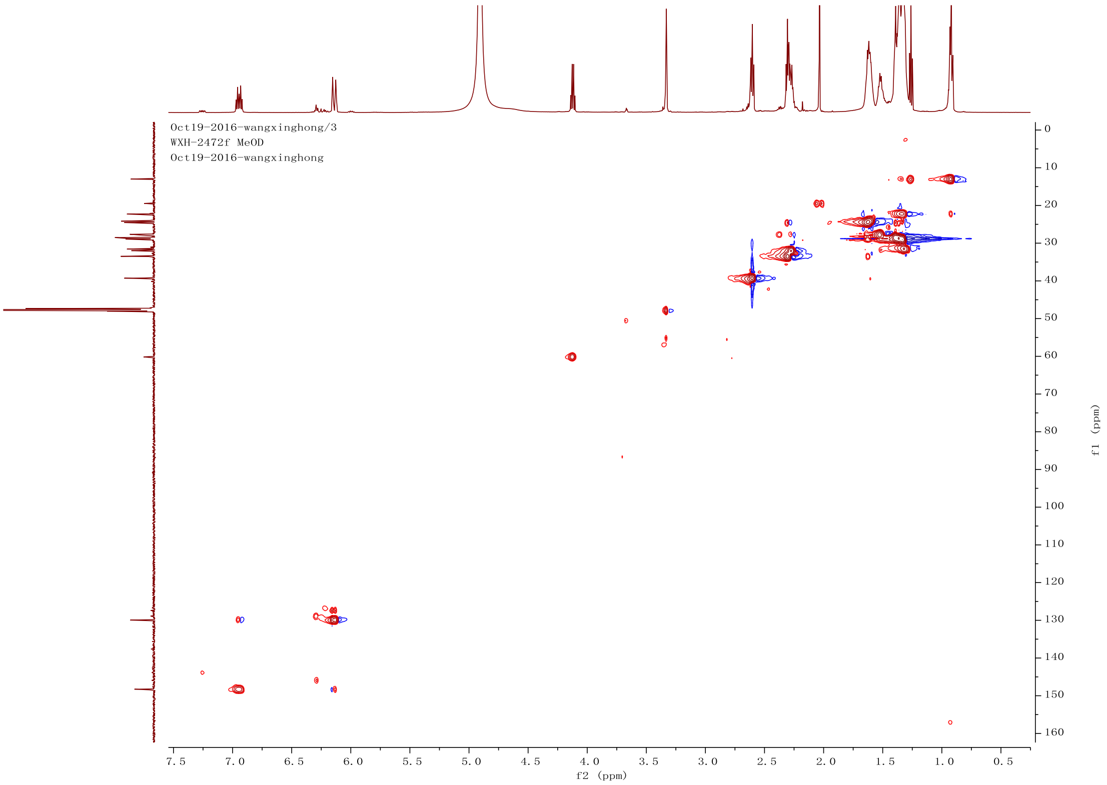

Supplementary Figure 12. HSQC of Mixture 2.

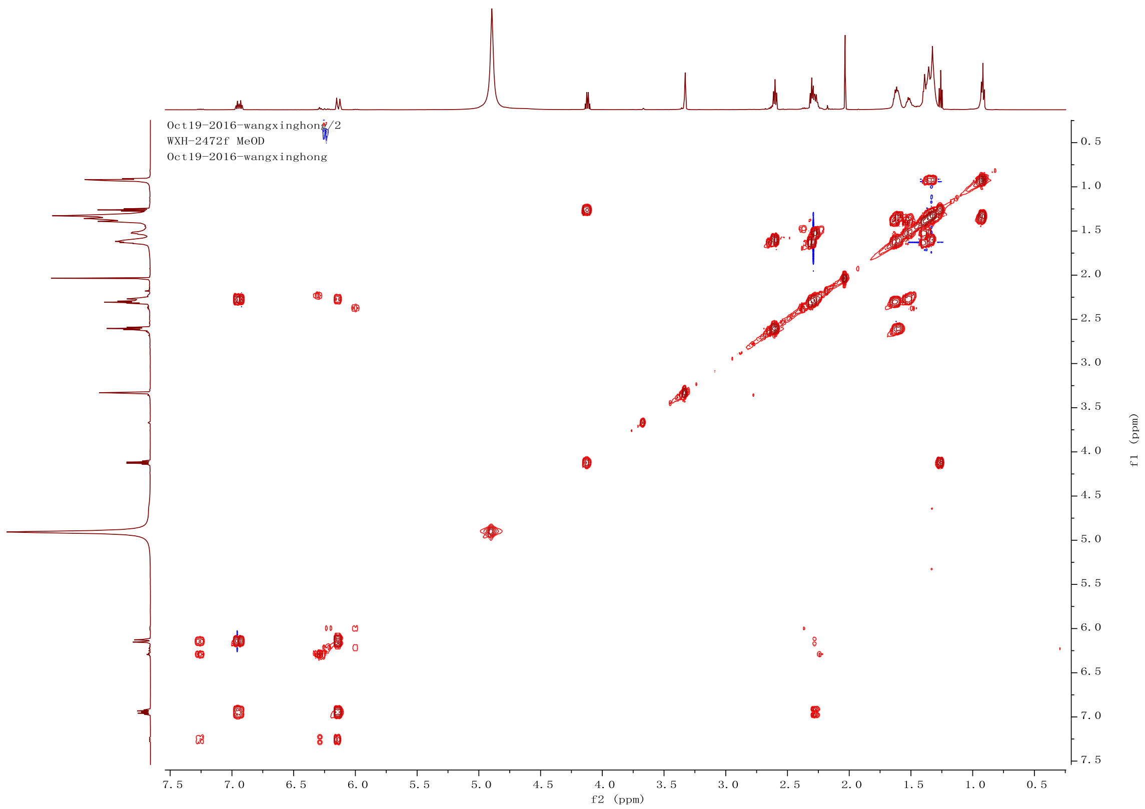

Supplementary Figure 13. 1H-1H COSY of Mixture 2.

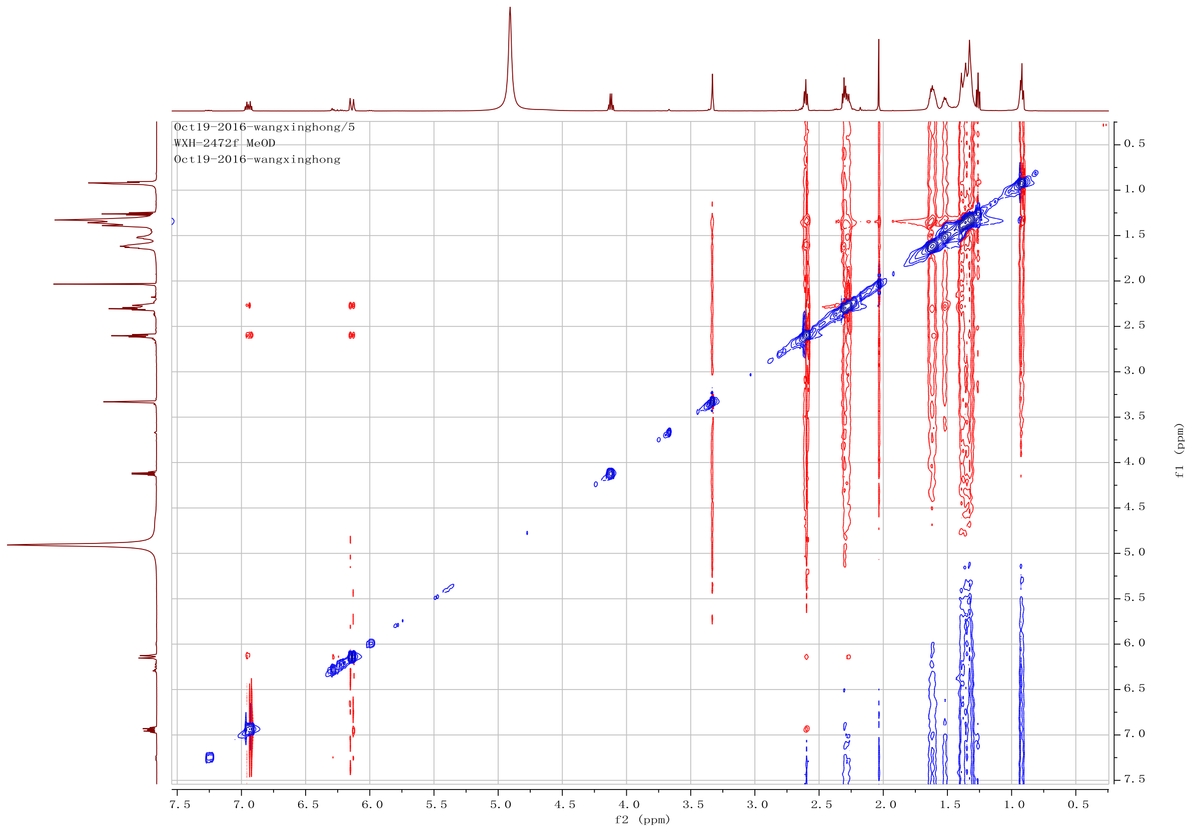

Supplementary Figure 14. ROESY of Mixture 2.

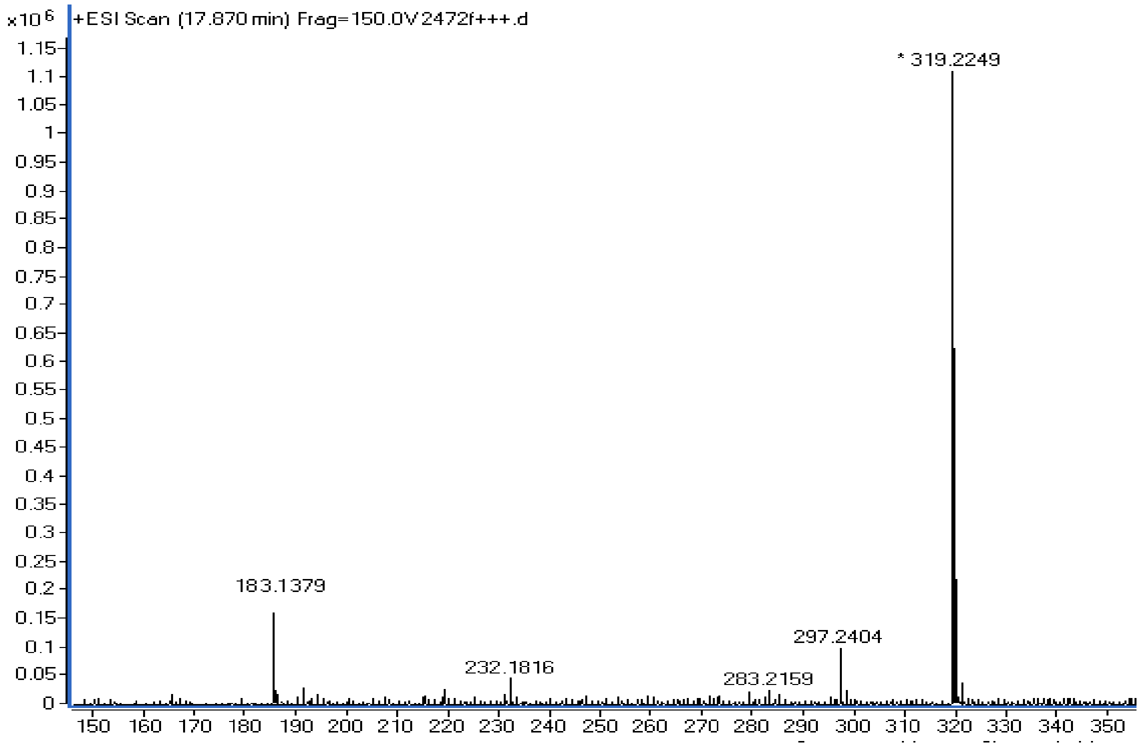

Supplementary Figure 15. The HR-ESI-MS of Compound 3.

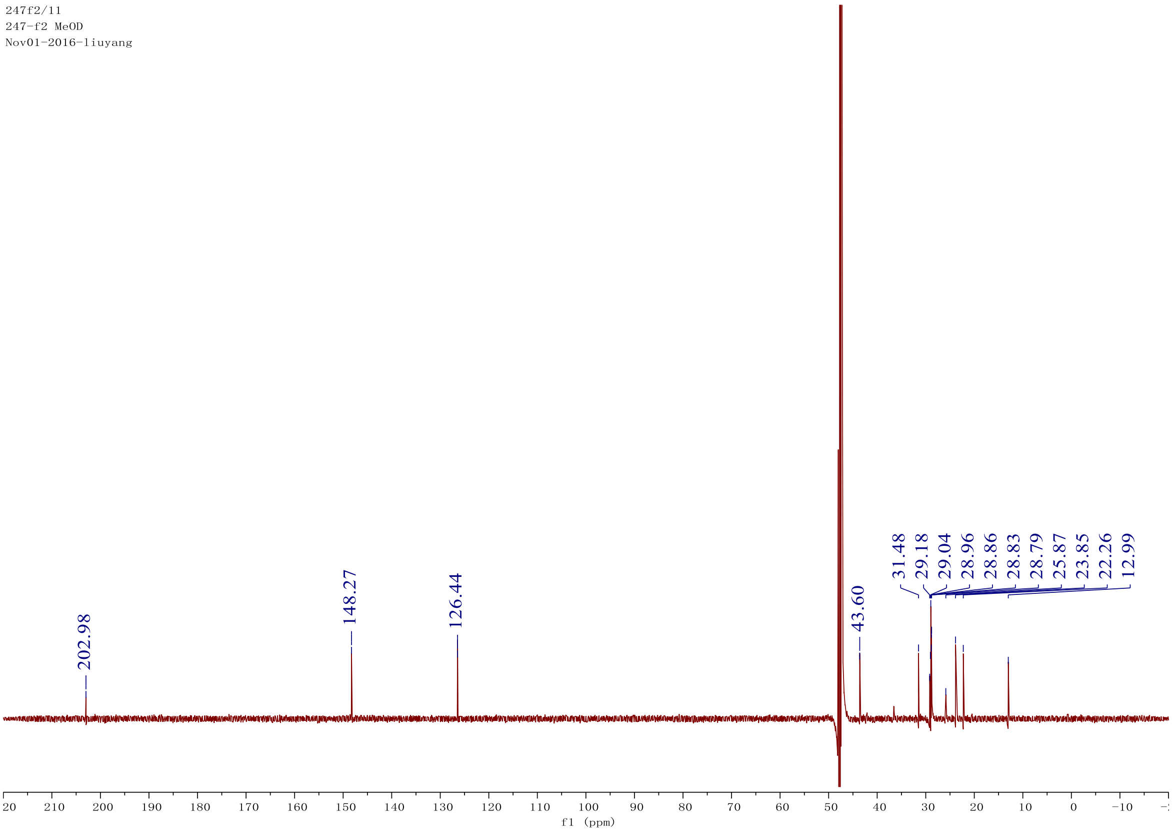

Supplementary Figure 16. 13C-NMR of Compound 3.

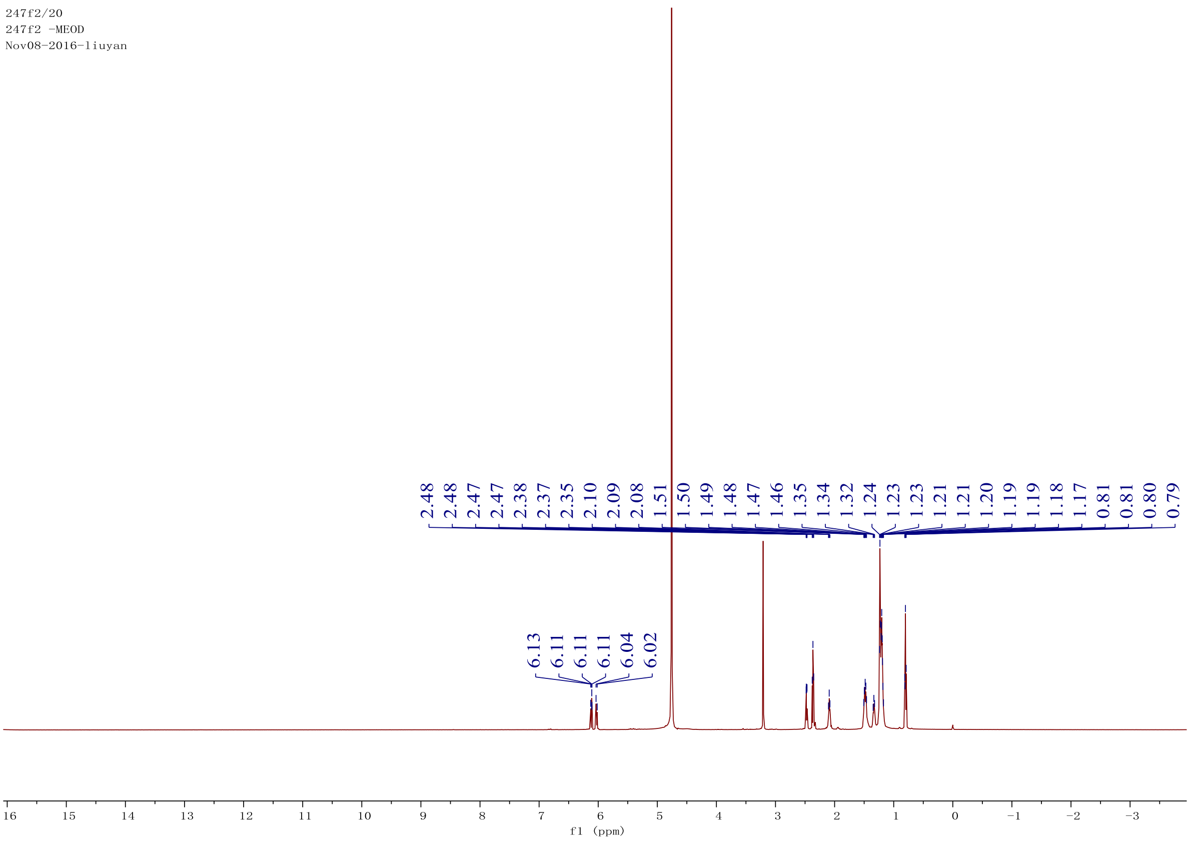

Supplementary Figure 17. 1H-NMR of Compound 3.

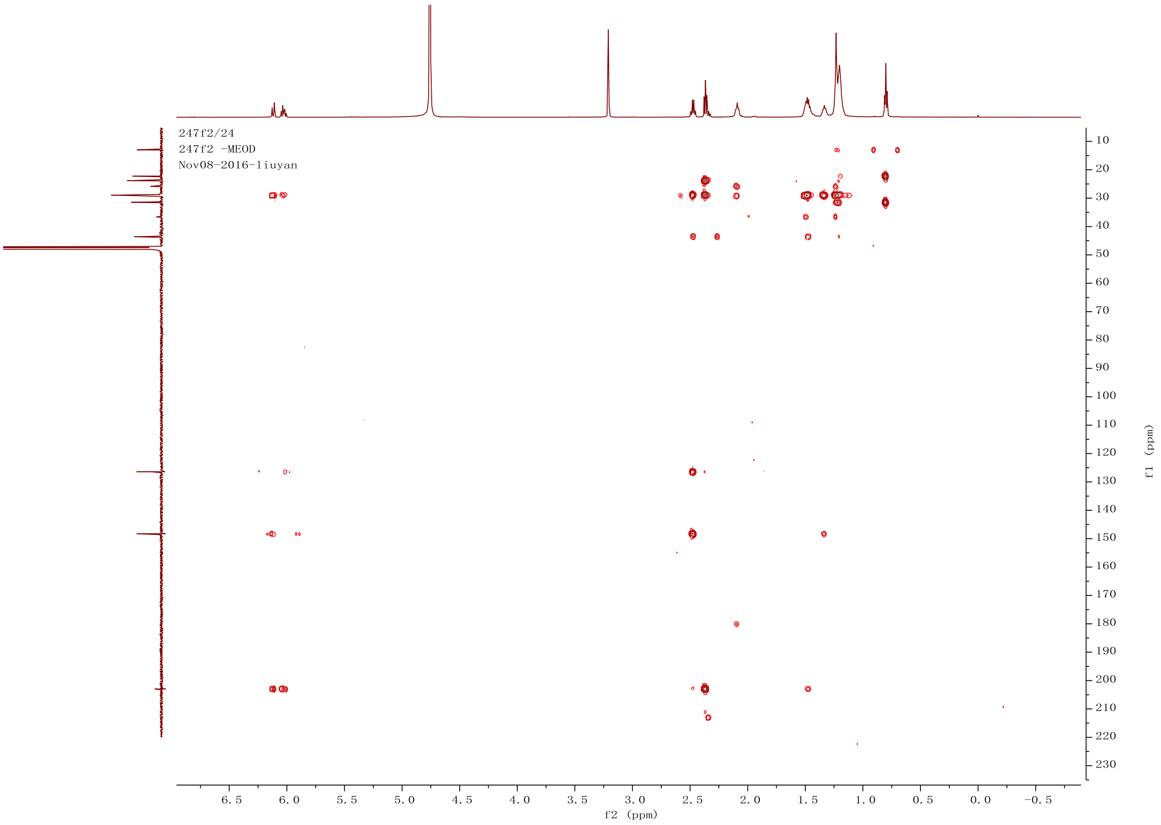

Supplementary Figure 18. HMBC of Compound 3.

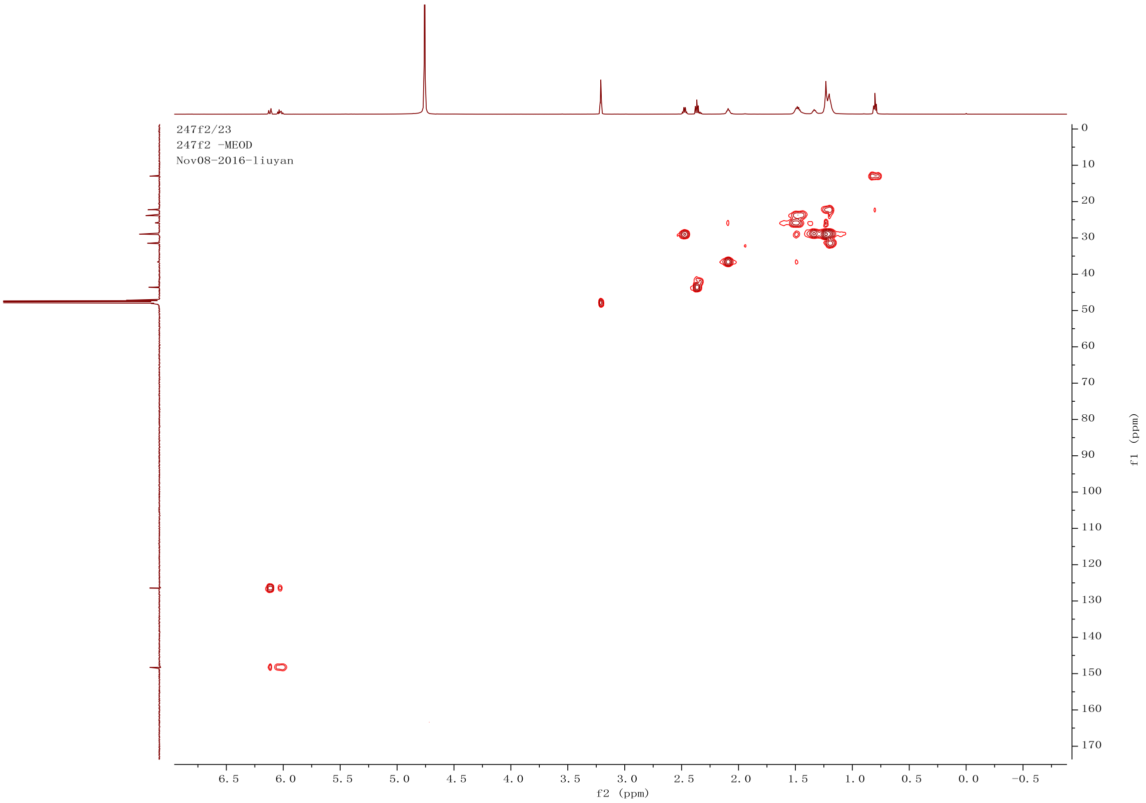

Supplementary Figure 19. HSQC of Compound 3.

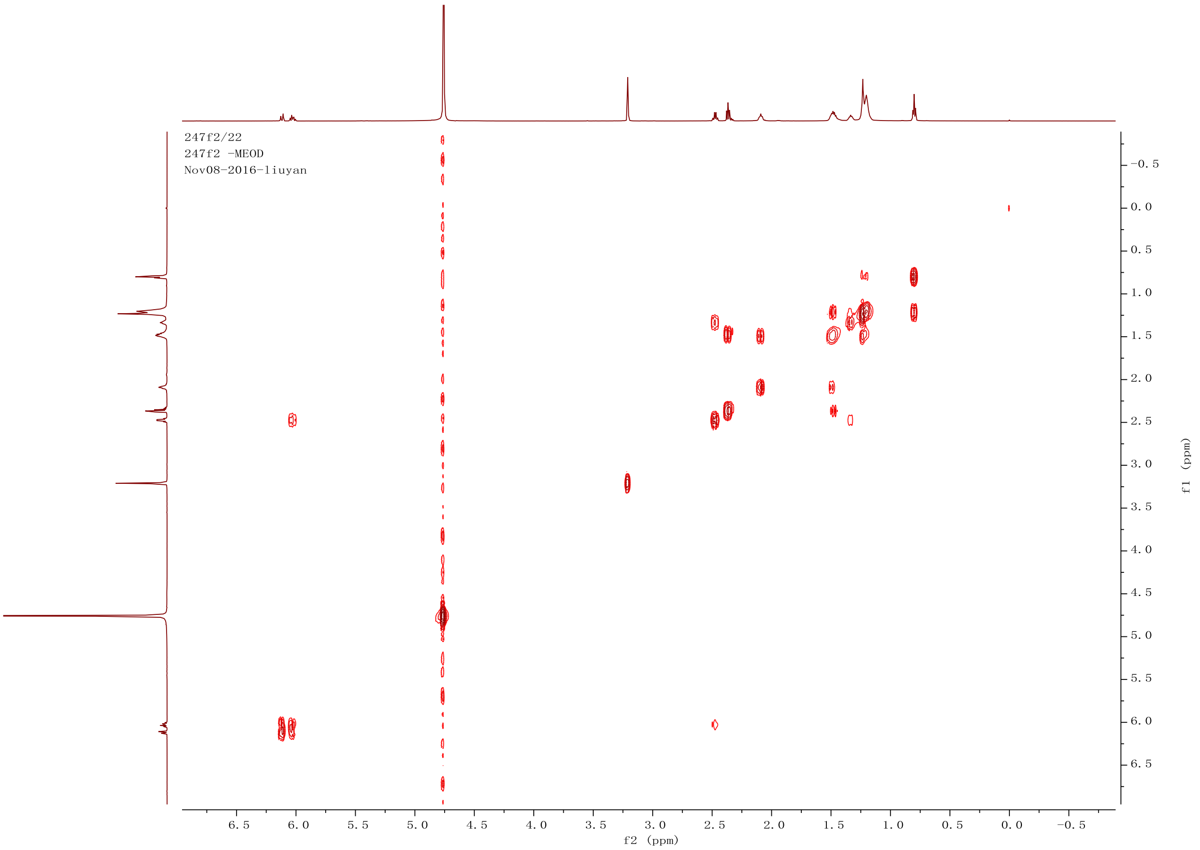

Supplementary Figure 20. S21 1H-1HCOSY of Compound 3.

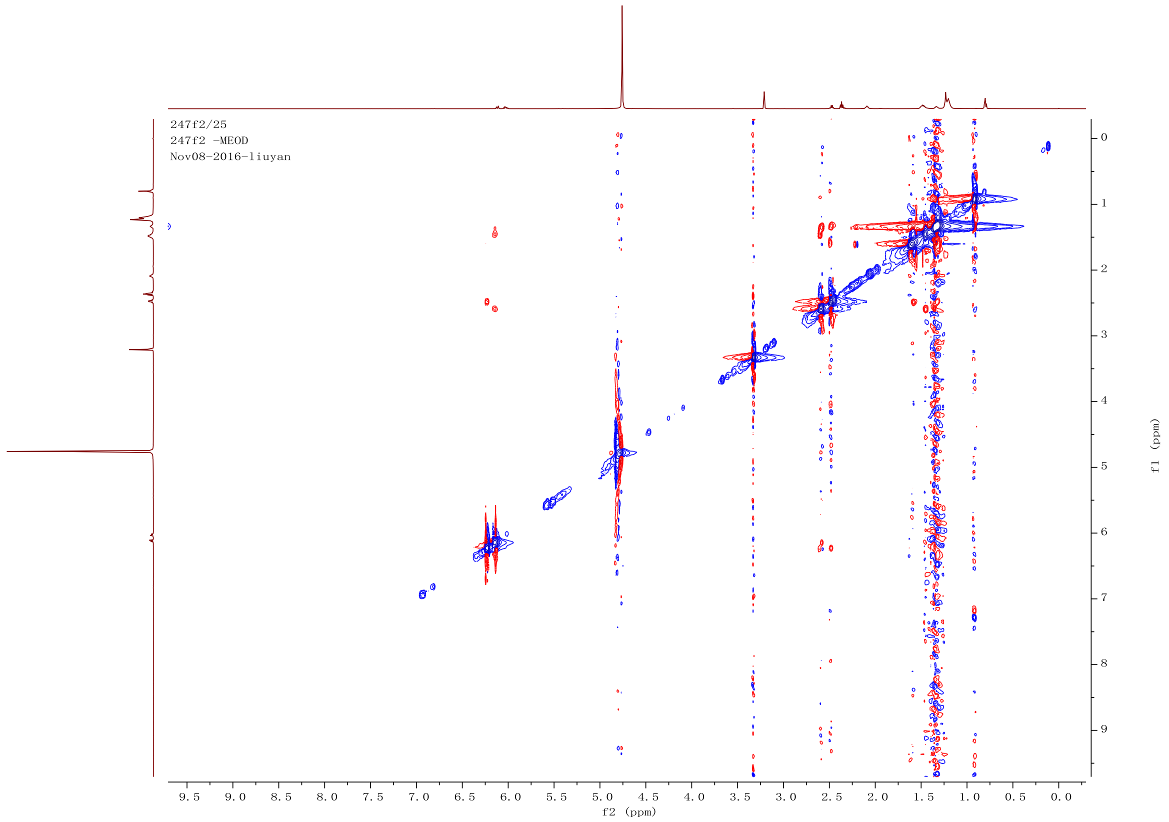

Supplementary Figure 21. ROESY of Compound 3.

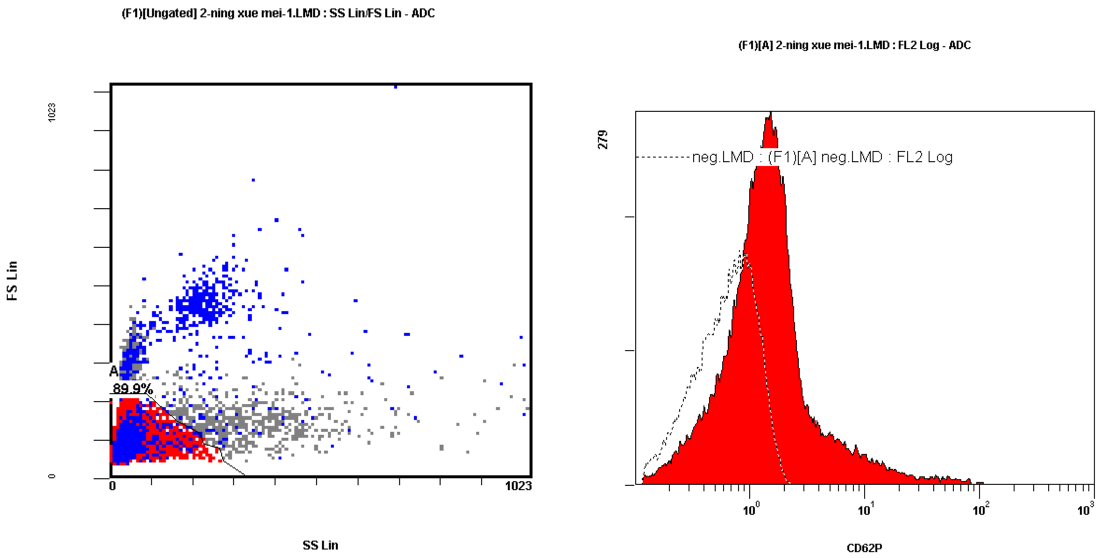

Supplementary Figure 22. Photos exemplifying the gating strategy of the flow cytometry for the platelet activation experiments. Instrument and software, Beckman FC-500, CXP Software.

Cell abundance: The amount of cells detected was about 10,000. CD62P antibody was used as

a marker. Gating technology: FSC/SSC was the most concentrated. Cells with similar diameter and particle size accounted for about 90% of all the collected cells. The others were cell debris and miscellaneous cells. Platelets were activated upon external stimulation. The activation degree was different in relation to different treatments. Double peak did not appear in the FACS image. The data used was the average fluorescence intensity.

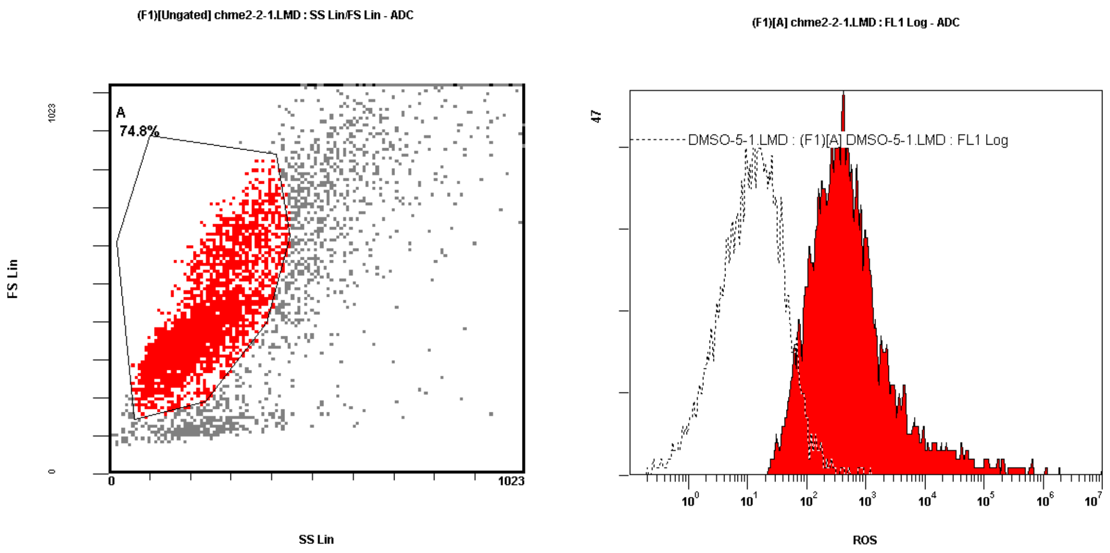

Supplementary Figure 23. Photos exemplifying the gating strategy for ROS determination. Instrument and software, Beckman FC-500, CXP Software.

Cell abundance: The amount of cells detected was about 10,000. MCF-7 cells were used for the measurement of ROS. Gating technology: FSC/SSC was the most concentrated. Cells with similar diameter and particle size accounted for about 75% of all the population. The others were cell debris and cells at a different growth stage. Double peak did not appear in the FACS image. The data used was the average fluorescence intensity.
