# Supplementary material for: Hydroxy-octadecenoic acids instead of phorbol esters are responsible for the Jatropha curcas kernel cake’s toxicity
Source: Commun Biol. 2020 May 8;3:228. doi: 10.1038/s42003-020-0919-z (PMC7210109; doi:10.1038/s42003-020-0919-z)
Supplement: Supplementary file 3 — Description of Additional Supplementary Files [file 42003_2020_919_MOESM3_ESM.pdf]

Description of Additional Supplementary Files

File Name: **Supplementary Data**

Description: The source data of all the graphic figures of the paper including Figures 5a, 6 and 7 are included in the respective Excel book of the “Supplementary Data”.
