# Supplementary material for: Hydroxy-octadecenoic acids instead of phorbol esters are responsible for the Jatropha curcas kernel cake’s toxicity
Source: Commun Biol. 2020 May 8;3:228. doi: 10.1038/s42003-020-0919-z (PMC7210109; doi:10.1038/s42003-020-0919-z)
Supplement: Supplementary file 4 — Reporting Summary [file 42003_2020_919_MOESM4_ESM.pdf]

nature research

Corresponding author(s): Changhe Zhang

Last updated by author(s): Mar 19, 2020

# Reporting Summary

Nature Research wishes to improve the reproducibility of the work that we publish. This form provides structure for consistency and transparency in reporting. For further information on Nature Research policies, see Authors & Referees and the Editorial Policy Checklist.

## Statistics

For all statistical analyses, confirm that the following items are present in the figure legend, table legend, main text, or Methods section.

| n/a | Confirmed | |
|---|---|---|
| ☐ | ☒ | The exact sample size ($n$) for each experimental group/condition, given as a discrete number and unit of measurement |
| ☐ | ☒ | A statement on whether measurements were taken from distinct samples or whether the same sample was measured repeatedly |
| ☐ | ☒ | The statistical test(s) used AND whether they are one- or two-sided<br>*Only common tests should be described solely by name; describe more complex techniques in the Methods section.* |
| ☒ | ☐ | A description of all covariates tested |
| ☐ | ☒ | A description of any assumptions or corrections, such as tests of normality and adjustment for multiple comparisons |
| ☐ | ☒ | A full description of the statistical parameters including central tendency (e.g. means) or other basic estimates (e.g. regression coefficient) AND variation (e.g. standard deviation) or associated estimates of uncertainty (e.g. confidence intervals) |
| ☒ | ☐ | For null hypothesis testing, the test statistic (e.g. $F$, $t$, $r$) with confidence intervals, effect sizes, degrees of freedom and $P$ value noted<br>*Give P values as exact values whenever suitable.* |
| ☒ | ☐ | For Bayesian analysis, information on the choice of priors and Markov chain Monte Carlo settings |
| ☒ | ☐ | For hierarchical and complex designs, identification of the appropriate level for tests and full reporting of outcomes |
| ☒ | ☐ | Estimates of effect sizes (e.g. Cohen's $d$, Pearson's $r$), indicating how they were calculated |

*Our web collection on statistics for biologists contains articles on many of the points above.*

## Software and code

Policy information about availability of computer code

| | |
|---|---|
| Data collection | No software was used |
| Data analysis | Excel 2016, Beckman Cytomics FC 500 CXP Software |

For manuscripts utilizing custom algorithms or software that are central to the research but not yet described in published literature, software must be made available to editors/reviewers. We strongly encourage code deposition in a community repository (e.g. GitHub). See the Nature Research guidelines for submitting code & software for further information.

## Data

Policy information about availability of data

All manuscripts must include a data availability statement. This statement should provide the following information, where applicable:

- Accession codes, unique identifiers, or web links for publicly available datasets
- A list of figures that have associated raw data
- A description of any restrictions on data availability

The authors declare that the main data supporting the findings of this study are available within the article and its Supplementary Information files. Source data for Figs. 5a and 6–7 are available in Supplementary Data. Extra data are available from the corresponding author upon reasonable request.

# Field-specific reporting

Please select the one below that is the best fit for your research. If you are not sure, read the appropriate sections before making your selection.

☒ Life sciences ☐ Behavioural & social sciences ☐ Ecological, evolutionary & environmental sciences

# Life sciences study design

All studies must disclose on these points even when the disclosure is negative.

| | |
|---|---|
| Sample size | Sample sizes were chosen on the basis of preliminary experiments so as to provide sufficient power for statistical comparison (where appropriate). |
| Data exclusions | No data were excluded for the analysis. |
| Replication | All replicates are reported in the manuscript. |
| Randomization | All groups were randomized. |
| Blinding | Blinding was not relavent to this study. |

# Reporting for specific materials, systems and methods

We require information from authors about some types of materials, experimental systems and methods used in many studies. Here, indicate whether each material, system or method listed is relevant to your study. If you are not sure if a list item applies to your research, read the appropriate section before selecting a response.

## Materials & experimental systems

| n/a | Involved in the study | |
|---|---|---|
| ☐ | ☒ | Antibodies |
| ☐ | ☒ | Eukaryotic cell lines |
| ☒ | ☐ | Palaeontology |
| ☐ | ☒ | Animals and other organisms |
| ☒ | ☐ | Human research participants |
| ☒ | ☐ | Clinical data |

## Methods

| n/a | Involved in the study | |
|---|---|---|
| ☒ | ☐ | ChIP-seq |
| ☐ | ☒ | Flow cytometry |
| ☒ | ☐ | MRI-based neuroimaging |

## Antibodies

| | |
|---|---|
| Antibodies used | AntiCD62P antibody, purchased from eBioscience, Lot Number 4318352. |
| Validation | Validation was performed by the manufacture. |

## Eukaryotic cell lines

Policy information about cell lines

| | |
|---|---|
| Cell line source(s) | LAD2, MCF-7 and L6 were purchased from Beina Biology, Beijing. |
| Authentication | Authentication was performed by the manufacture. |
| Mycoplasma contamination | All cell lines were tested negative for mycoplasma contamination. |
| Commonly misidentified lines (See ICLAC register) | No misidentified cell lines were used. |

## Animals and other organisms

Policy information about studies involving animals; ARRIVE guidelines recommended for reporting animal research

| | |
|---|---|
| Laboratory animals | Kunming mice, rats, Guinea pigs and carp fingerlings. |
| Wild animals | This study did not involve wild animals. |
| Field-collected samples | This study did not involve field-collected samples. |
| Ethics oversight | The Commission on Animal Health and Ethics of Yunnan University, Kunming, China. |

Note that full information on the approval of the study protocol must also be provided in the manuscript.

# Flow Cytometry

## Plots

Confirm that:

☒ The axis labels state the marker and fluorochrome used (e.g. CD4-FITC).

☒ The axis scales are clearly visible. Include numbers along axes only for bottom left plot of group (a 'group' is an analysis of identical markers).

☐ All plots are contour plots with outliers or pseudocolor plots.

☒ A numerical value for number of cells or percentage (with statistics) is provided.

## Methodology

| | |
|---|---|
| Sample preparation | The platelet and MCF-7 cell samples were prepared as described in the manuscript. |
| Instrument | Beckman FC/500 |
| Software | CXP software of Beckman |
| Cell population abundance | The amount of cells detected was about 10,000. |
| Gating strategy | Determination of rat platelet CD62P activity: FSC/SSC was the most concentrated. Cells with similar diameter and particle size accounted for about 90% of all the collected cells. The others were cell debris and miscellaneous cells. Platelets were activated upon external stimulation. The activation degree was different in relation to different treatments. Double peak did not appear in the FACS image. The data used was the average fluorescence intensity.<br><br>Measurements of reactive oxygen species (ROS) in MCF-7 cells: FSC/SSC was the most concentrated. Cells with similar diameter and particle size accounted for about 75% of all the population. The others were cell debris and cells at a different growth stage. Double peak did not appear in the FACS image. The data used was the average fluorescence intensity (Supplementary Fig. 23). |

☒ Tick this box to confirm that a figure exemplifying the gating strategy is provided in the Supplementary Information.
